# Prognostic roles of tumor associated macrophages in bladder cancer: a system review and meta-analysis

**DOI:** 10.18632/oncotarget.25334

**Published:** 2018-05-18

**Authors:** Shui-Qing Wu, Ran Xu, Xue-Feng Li, Xiao-Kun Zhao, Bin-Zhi Qian

**Affiliations:** ^1^ Department of Urology, The Second Xiangya Hospital, Central South University, 410011, Hunan Province, People's Republic of China; ^2^ MRC Centre for Reproductive Health, EH16 4TJ, Edinburgh, United Kingdom; ^3^ Edinburgh Cancer Research UK Centre Queen's Medical Research Institute, EH16 4TJ, Edinburgh, United Kingdom

**Keywords:** tumor associated macrophages, bladder cancer, system review, meta-analysis

## Abstract

**Background:**

Tumor associated macrophages (TAMs) have multifaceted roles in the development of many tumor types. However, the prognostic value of TAMs in bladder cancer is still not conclusive.

**Experimental design:**

This review evaluated the prognostic value of TAMs density in bladder cancer by reviewing published literatures and integrating the results via a meta-analysis. A systematic search was conducted in PubMed, Embase and Chinese National Knowledge Infrastructure (CNKI), WanFang, and Web of Science databases for relevant studies. Overall survival (OS), relapse free survival (RFS), disease specific survival (DSS), and progression free survival (PFS) were assessed in bladder cancer patients.

**Results:**

The pooled hazard ratios (HRs) and 95% confidence intervals (CIs) indicated that TAMs identified with CD68 alone have no significant correlation with OS (HR = 1.01, 95% CI = 1.00–1.02), RFS (HR = 0.99, 95% CI = 0.91–1.06), or PFS (HR = 1.19, 95% CI = 0.70–1.68) in bladder cancer patients. Subgroup analyses involved with Bacillus Calmette Guerin (BCG) treatment or sample locations either showed that CD68^+^ TAMs presented no prognostic value with regard to OS in bladder cancer patients. However, TAMs detected by CD163 are significantly correlated with poor RFS in bladder cancer patients (HR = 1.54, 95% CI = 1.16–1.92).

**Conclusions:**

Our data indicated that TAMs identified only with CD68 have no significant correlation with the prognosis and clinicopathological parameters of bladder cancer patients. However, TAMs detected with CD163 could serve as a prognostic marker for bladder cancer patients. These findings invite further research on the role of TAM subsets in bladder cancer patients.

## INTRODUCTION

Bladder cancer is one of the most common malignant tumors in urological diseases. In USA alone, 58950 new cases of bladder cancer and 11820 cases bladder cancer related deaths were estimated in 2016. These accounted for 7% of all new cancer diagnoses and 4% of all cancer deaths [1]. Transurethral resection of bladder tumor (TURBT) or radical cystectomy (RC) is the primary therapy for NMIBC or MIBC respectively. Following TURBT, bladder instillation of drugs was taken as effective choice to prevent or reduce the recurrence, especially for intermediate and high risk bladder tumors. Many kinds of drugs were recommended to be in use after TURBT, such as chemotherapy and immunotherapy drugs [2]. Bacillus Calmette-Guerin (BCG) intravesical immunotherapy is a classic and effective therapy to inhibit the relapse of intermediate and high risk non-muscle-invasive bladder cancer (NMIBC). However, around 39% of NMIBC patients still relapse and 8% will progress to muscle-invasive bladder cancer (MIBC) [3–5]. High recurrence rate of NMIBC results in repeated TURBT, which leads to huge physical and economical burden to the patients. Hence, it is critical to explore and validate new prognostic biomarkers, which may be helpful for the treatment decisions and the management of bladder cancer.

Macrophages are important regulators in both tissues homeostasis and tumor progression. Increasing evidences have shown that tumor associated macrophages (TAMs) are involved in tumor initiation, progression, angiogenesis, drugs resistance and metastasis. Different phenotypes of macrophages often present different roles in tumor microenvironment, which are tightly regulated by factors in the tumor microenvironment including interleukin-4, interleukin-10, immunoglobulin etc. [6].

Several recent studies have investigated the prognostic role of TAMs in different tumors [7, 8]. In many cancer types (such as gastric cancer, breast cancer, thyroid cancer), high density of TAMs is correlated with poor prognosis. However, in colorectal cancer high density of TAMs is correlated with good prognosis [7–9]. Moreover, the prognostic role of TAMs in specific cancer type may vary depending on different biomarkers used for TAM detection or quantification in various parts of the samples [8, 9]. As for bladder cancer, the prognostic role of TAMs in bladder cancer remains controversial among different studies [10–22]. To address this controversy, we performed this meta-analysis based on the TAMs identified by CD68 and CD163 markers to evaluate the potential association between TAMs density and prognostic outcomes in bladder cancer.

## RESULTS

### Search results and characteristics of studies

**Figure 1:**
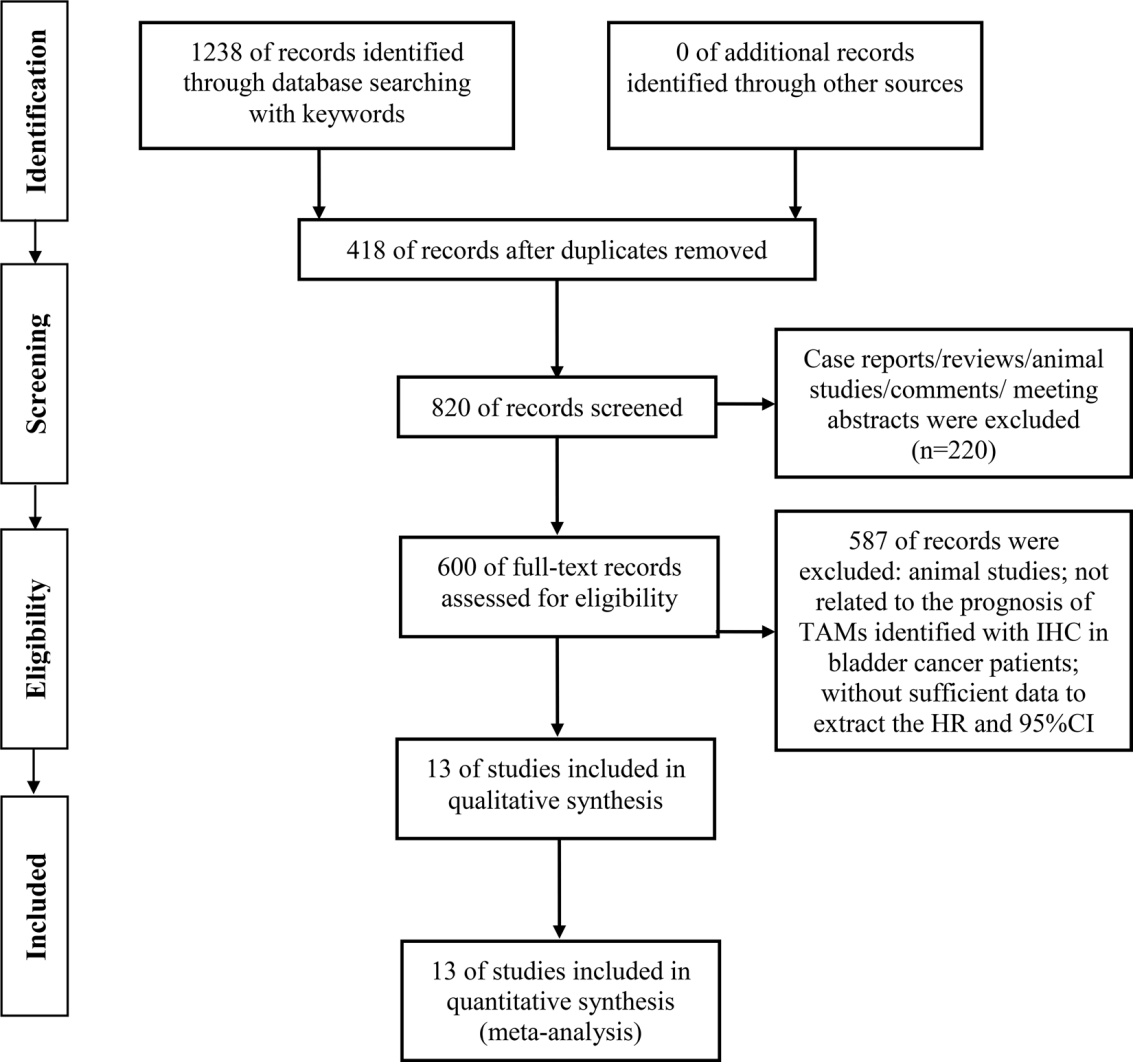
Flow diagram for the selection of included studies

1238 records were identified from the different databases with keywords and search strategy detailed in methods. 418 duplicate records were removed by the detection of Endnote X7 software. The remaining records were screened by the titles and abstracts, and 220 records were excluded for case reports, reviews, letters, comments or meeting abstract. 600 remaining studies were further evaluated for eligibility, and 587 studies were further excluded for the following reasons: animal studies, not relevant with prognosis of TAMs identified with IHC in bladder cancer patients, without sufficient data to extract the HRs and 95% CIs. Based on the inclusion and exclusion criteria detailed in methods, 13 studies with macrophages detected by immunohistochemistry (IHC) in tumor samples were included for meta-analysis [10–22]. The flow chart of search strategy was shown in Figure 1.

12 of 13 included studies were published in English, while 1 study was published in Chinese. The total sample size for all records was 1400 cases (range from 27 to 337). Among these cases, 294 of them underwent BCG treatment. Of the 13 studies, 8 studies detected TAMs in whole sample area (or not specified) [10, 13–17, 21, 22], 2 studies were involved with TAMs detected in tumor islet and tumor stroma, respectively [19, 20], 1 study focused TAMs locating in tumor stroma [18], 1 study reported TAMs detected in tumor islet, tumor stroma and whole sample area, respectively [12], and 1study investigated macrophages identified in invasive front or middle to superficial carcinoma parts separately [11]. OS, RFS, DSS and PFS were used jointly or separately as prognostic endpoints in the included studies. Details were depicted in Table 1 and the mean Newcastle-Ottawa Scale (NOS) scores of publication quality were 7.54.

**Table 1:** The characteristics of included studies

Study, Year	Country	Cases number	Tumor stages	Therapy	Biomarkers	Antibody Source	Sample locations	Follow up (midian months)	Outcome	NOS score
Hanada T, 2000	Japan	63	T_a_, T_1-4_	TUR, RC	CD68	Anti-CD68, mAb, Dako, Glostrup, Denmark	W	65 (3–153)	OS	7
Koga F, 2004	Japan	69	T_1_, T_2-4_	RC	CD68	Anti-CD68, mAb, KP1, Dako, Carpenteria, California, dilution 1:200	I^*^, M	58 (2–196)	DSS	7
Takayama H, 2009	Japan	44	Tis	BCG	CD68	Anti-CD68, mAb, DACO JAPAN, Kyoto, Japan	I, S, W	55.2 (3–240.5)	RFS	9
Ayari C, 2009	Canada	46	T_a_, T_1_, Tis	TUR+BCG	CD68	Anti-CD68, CloneKP1, DAKO, Glostrup, Denmark	W	26	RFS	8
Ajili F, 2013	Tunisia	27	T_a_, T_1_	TUR+BCG	CD68	Anti-CD68, CloneNCL-L-CD68, Leica, dilution 1:40	W	26	RFS	5
Suriano F, 2013	Italy	40	NMIBC	TUR+BCG	CD68, CD163	Anti-CD68, mAb, PG-M1; Anti-CD163, mAb; dilution 1:200	W	NR	RFS	6
Ayari C, 2013	Canada	93	T_a_, T_1_	TUR	CD68	Anti-CD68, cloneKP1 from DAKO, Glostrup, Denmark, dilution 1:400	W	68.4	RFS	8
Sjödahl G, 2014	Sweden	52	MIBC	RC	CD68	Anti-CD68, cloneEBM11 from Dako, dilution1:1500	W	70	DSS, PFS	8
Lima L, 2014	Portugal	99	T_a_, T_1_	TUR+BCG	CD163	Anti-CD163, mAb, Clone10D6; Novocastra-Leica, dilution 1:100	S	97 (13–163)	RFS	8
Wang B, 2015	China	302	T_a_, T_1-4_	TUR, RC	CD68	Anti-CD68, DakoA/S, Glostrup, Copenhagen, Denmark, dilution1:500	I, S	82 (4–137)	OS, RFS	8
Shao J, 2015	China	337	T_1_	TUR+THP	CD163	Anti-163, 10D6, Maixin, Fuzhou, China	I, S	32.4 (2.4–111.6)	RFS	7
Boström MM, 2015	Finland	184	T_a_, Tis, T_1-4_	TUR, RC	CD68	Anti-68, mAb, ab845, Abcam, U.K, dilution 1:5	W	6.9 (TUR); 4.2 (RC)	OS, RFS, DSS, PFS	9
Pichler R, 2016	Austria	40	T_a_, T_1_, Tis	TUR+BCG	CD68, CD163	Anti-163, mAb, CloneMRQ-26, prediluted, Roche; Anti-68, mAb, ClonePG-M1, Dako, dilution 1:50	W	29.5	RFS	8

Abbreviations: TUR: transurethral resection; RC: radical cystectomy; BCG: Bacillus Calmette Guerin vaccine; THP: Therarubicin; S: Stroma; I: Intratumoral or Islet; I^*^: invasive front; M: middle to superficial carcinoma parts; W: whole sample area (or not specified); OS: overall survival; RFS: relapse free survival; CSS: cancer specific survival.

### Detection of tumor associated macrophages

CD68 was used for identification of TAMs in 12 of the 13 included studies, including 2 studies in combination with CD163 [15, 22]. Both CD68 and CD163 biomarkers were identified by immunohistochemical staining in all the included studies. The antibodies are listed in Table 1. The prognostic role of CD68^+^ TAMs on RFS was assessed in 7 studies [12–16, 21, 22], of which 2 studies calculated CD68^+^ TAMs in different locations [12, 19]. The prognostic role of CD68^+^ TAMs on DSS was assessed in 3 studies, of which 1 study investigated the correlation of TAMs with DSS in two populations of patients treated by TUR or RC, and Koga F's study analyzed the association between DSS and TAMs identified in different locations [11, 17, 21]. The prognostic role of CD68^+^ TAMs on OS was evaluated in 3 studies, of which 1 study evaluated the relationship between CD68^+^ TAMs and OS in TUR or RC population, respectively [10, 19, 21]. Moreover, the prognostic role of CD68^+^ TAMs on PFS was assessed in 2 studies [17, 21]. The prognostic role of TAMs identified with CD163 on RFS was evaluated in 4 studies, of which 1 study assessed the correlation of RFS with CD163 detected in tumor stroma or islet, respectively [15, 18, 20, 22].

### The association between CD68^+^ TAMs and prognostic outcomes

**Figure 2:**
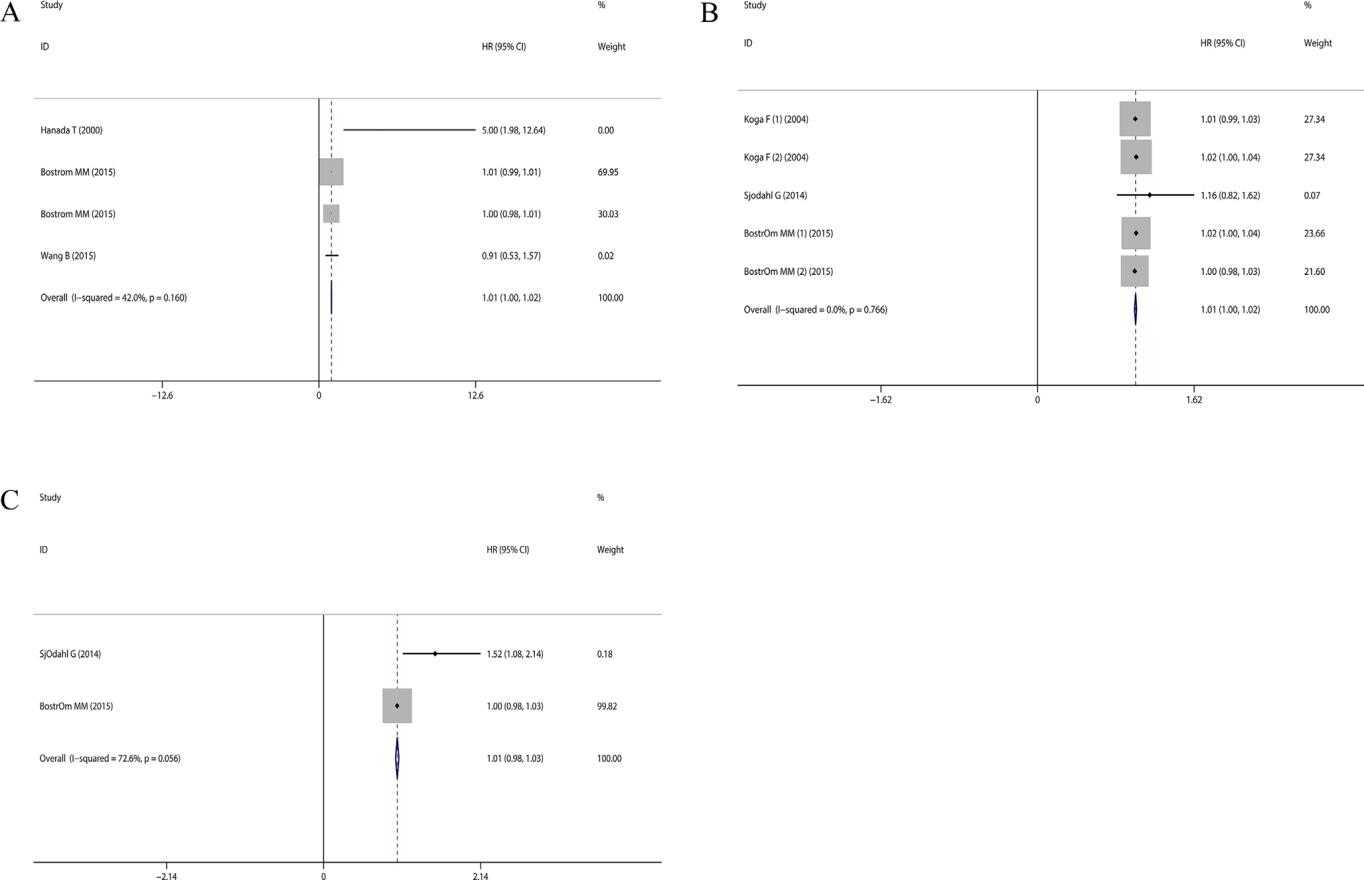
Forest plots evaluating the prognostic role of CD68^+^ TAMs in bladder cancer patients (**A**) CD68^+^ TAMs with OS; (**B**) CD68^+^ TAMs with DSS; (**C**) CD68^+^ TAMs with PFS.

Although Hanada T, et al [10] reported that higher CD68^+^ TAMs density significantly correlated with decreased OS (HR = 5.0, 95% CI = 1.98 ~ 12.64), the pooled HR concerning the prognostic role of CD68^+^ TAMs on OS was 1.01 (95% CI = 1.00 ~ 1.02) (Figure 2A), which indicated the prognostic role of CD68^+^ TAMs should be cautiously interpreted in bladder cancer patients.

**Figure 3:**
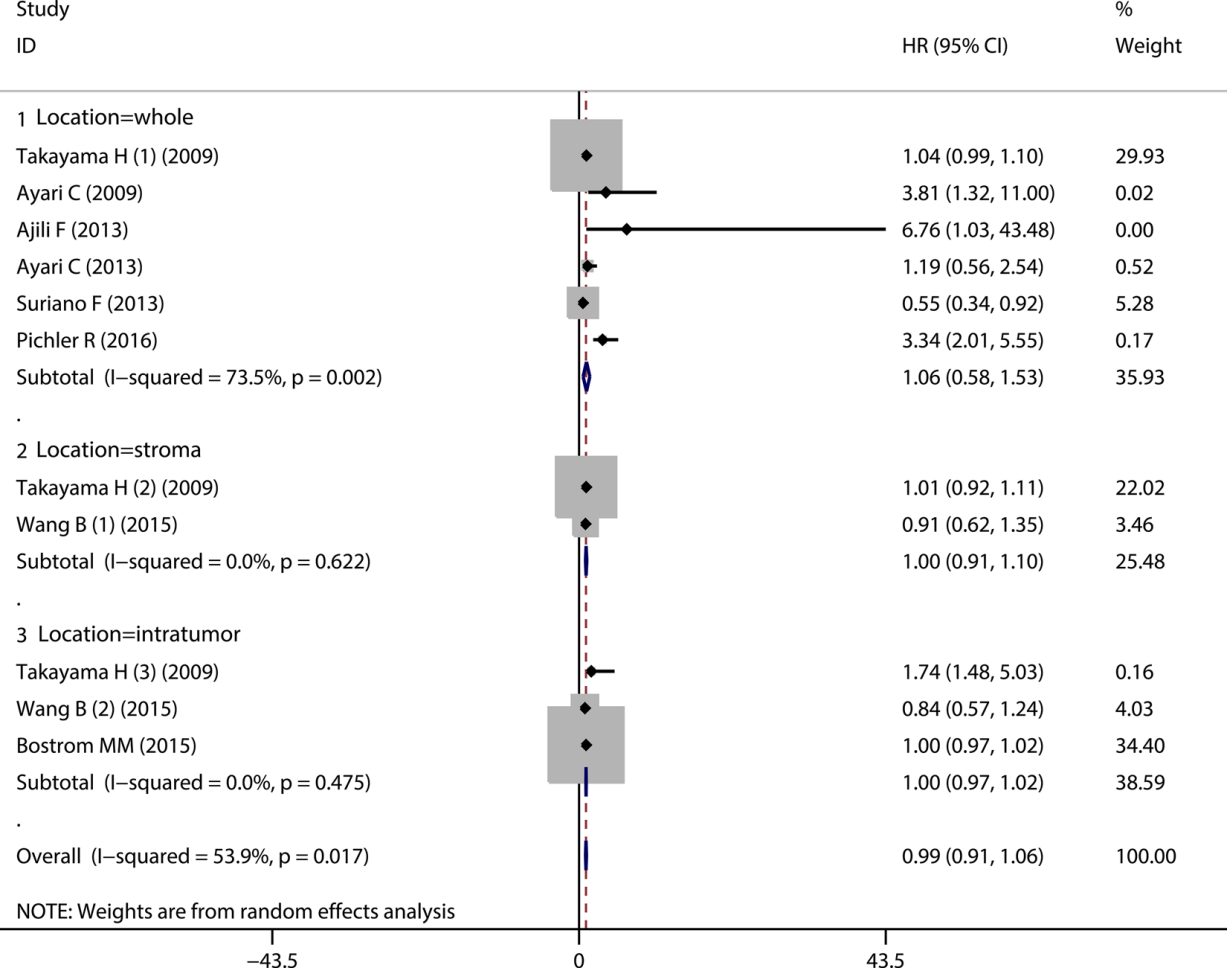
Forest plot evaluating the prognostic role of CD68^+^ TAMs on RFS in bladder cancer patients Subgroup analyses with regard to different sample locations.

As for the prognostic value of CD68^+^ TAMs on RFS, the pooled HR showed that elevated CD68^+^ TAMs density was not significantly associated with the RFS (HR = 0.99, 95% CI = 0.91 ~ 1.06) (Figure 3, Overall). Furthermore, subgroup analyses according to the CD68^+^ TAMs in different sample locations also showed that the high CD68^+^ TAMs infiltration presented no significant correlation with RFS in bladder cancer patients with regard to whole sample area (or not specified) (HR = 1.06, 95% CI = 0.58 ~ 1.53) (Figure 3, Location = whole), tumor stroma (HR = 1.00, 95% CI = 0.91 ~ 1.10) (Figure 3, Location = stroma) and tumor islet (HR = 1.00, 95% CI = 0.97 ~ 1.02) (Figure 3, Location = intratumor). Similarly, subgroup analyses according to the history of BCG treatment showed that the pooled HRs for RFS in the patients with or without BCG therapy were 0.96 (95% CI = 0.79 ~ 1.14) and 0.99 (95% CI = 0.97 ~ 1.02), respectively (Supplementary Figure 1). Collectively, no significant correlation between CD68^+^ TAMs and RFS could be found in bladder cancer patients.

To further investigate the prognostic role of CD68^+^ TAMs on DSS, the pooled HRs demonstrated no significant association between CD68^+^ TAMs and DSS in bladder cancer patients (HR = 1.01, 95% CI = 1.00 ~ 1.02) (Figure 2B). Moreover, the pooled results based on meta-analysis also indicated that there was no significant association between CD68^+^ TAMs and PFS in bladder cancer patients (HR = 1.19, 95% CI = 0.70~1.68) (Figure 2C). Taken together, no significant association could be found between CD68^+^ TAMs and OS, RFS, DSS and PFS of bladder cancer patients in our meta-analysis.

### The association of CD68^+^ TAM with clinicopathological characteristics

We further evaluated the role of elevated TAMs with the clinicopathological parameters in bladder cancer patients. Four of the 13 included studies have presented relevant data of the association between clinicopathological parameters and CD68^+^ TAMs, while there was not enough data for CD163^+^ TAMs. Pooled analysis showed no significant correlation between elevated CD68^+^ TAMs infiltration and sex (Male vs. Female), sge (≥ 70 years VS < 70 years), carcinoma *in situ* (yes vs. no), tumor stage (T1 vs. Ta), tumor grade (≥ 2 vs. 1) or tumor size (> 3 cm vs. ≤ 3 cm) (detailed in Table 2).

**Table 2:** The evaluation of elevated CD68^+^ macrophages with clinicopathological characteristics

Patient charateristics	References of studies	Number of patients	Effect models	OR (95% CI)	Heterogeneity	
					I^2^ (%)	P_h_
Sex (Male vs. Female)	11, 14, 17	202	fixed	0.47 (–0.10–1.039)	0	0.91
Age (≥ 70 years VS < 70 years)	14, 17	139	fixed	0.76 (–0.07–1.59)	1	0.315
carcinoma *in situ* (yes vs. no)	14, 15	73	fixed	1.74 (–3.31–6.79)	0	0.50
Tumor stage (T1 vs. Ta)	14, 15, 17	166	fixed	2.77 (–0.72–6.26)	0	0.885
Number of tumor (> 1 vs. 1)	14, 15, 17	166	fixed	1.24 (0.11–2.37)	0	0.90
Tumor grade (≥ 2 vs. 1)	11, 14, 17	202	fixed	1.90 (–0.38–4.18)	0	0.91
Tumor size (> 3 cm vs. ≤ 3 cm)	14, 15	73	fixed	1.17 (–0.51–2.86)	0	0.39

### Prognostic significance of CD163^+^ TAMs in bladder cancer patients

**Figure 4:**
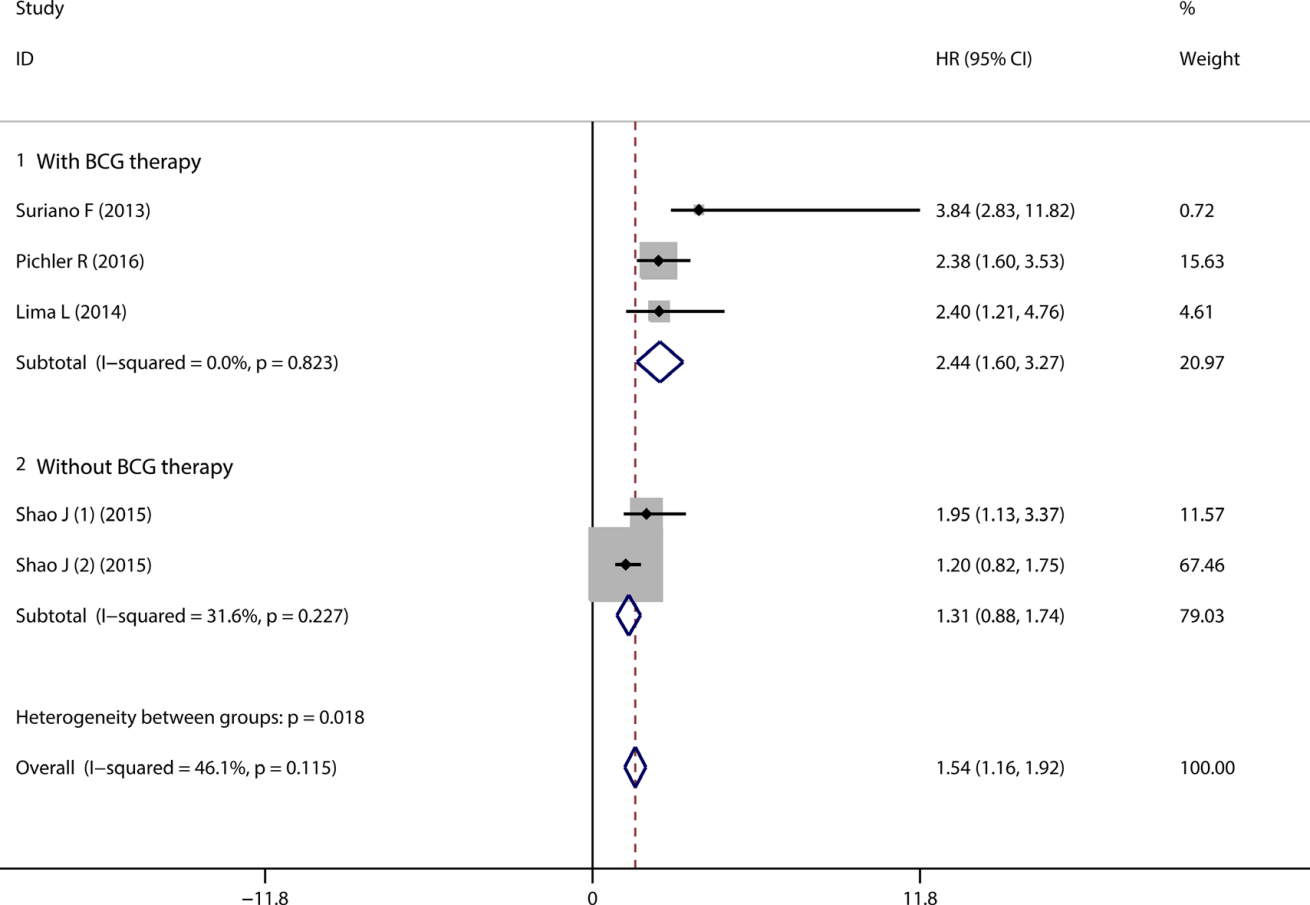
Forest plot of pooled HR for CD163^+^ TAMs on RFS in bladder cancer patients Subgroup analyses with regard to the history of BCG therapy.

4 of the 13 studies reported the correlation of CD163^+^ TAMs infiltration with RFS in bladder cancer patients who underwent TUR therapy. 3 of the 4 studies reported the patients received BCG therapy after TUR [16, 19, 23]. The pooled HR revealed that high density of TAMs was correlated with worse RFS (HR = 1.54, 95% CI = 1.16 ~ 1.92) (Figure 4, Overall), without significant heterogeneity among studies (*P* = 0.115, I^2^ = 46.1%). Subgroup analysis according to the history of BCG therapy indicated that high density of CD163^+^ TAMs infiltration correlated with worse RFS in patients who underwent BCG therapy after TUR (HR = 2.44, 95% CI = 1.60 ~ 3.27) (Figure 4, with BCG therapy). Subgroup analysis according to the sample locations suggested that elevated CD163^+^ TAMs density in both total tumor and tumor stroma correlated with unfavourable RFS (HRs = 2.44, 95% CI = 1.50 ~ 3.39, and = 2.08, 95% CI = 1.13 ~ 3.02, respectively) (Supplementary Figure 2).

### Publication bias

**Figure 5:**
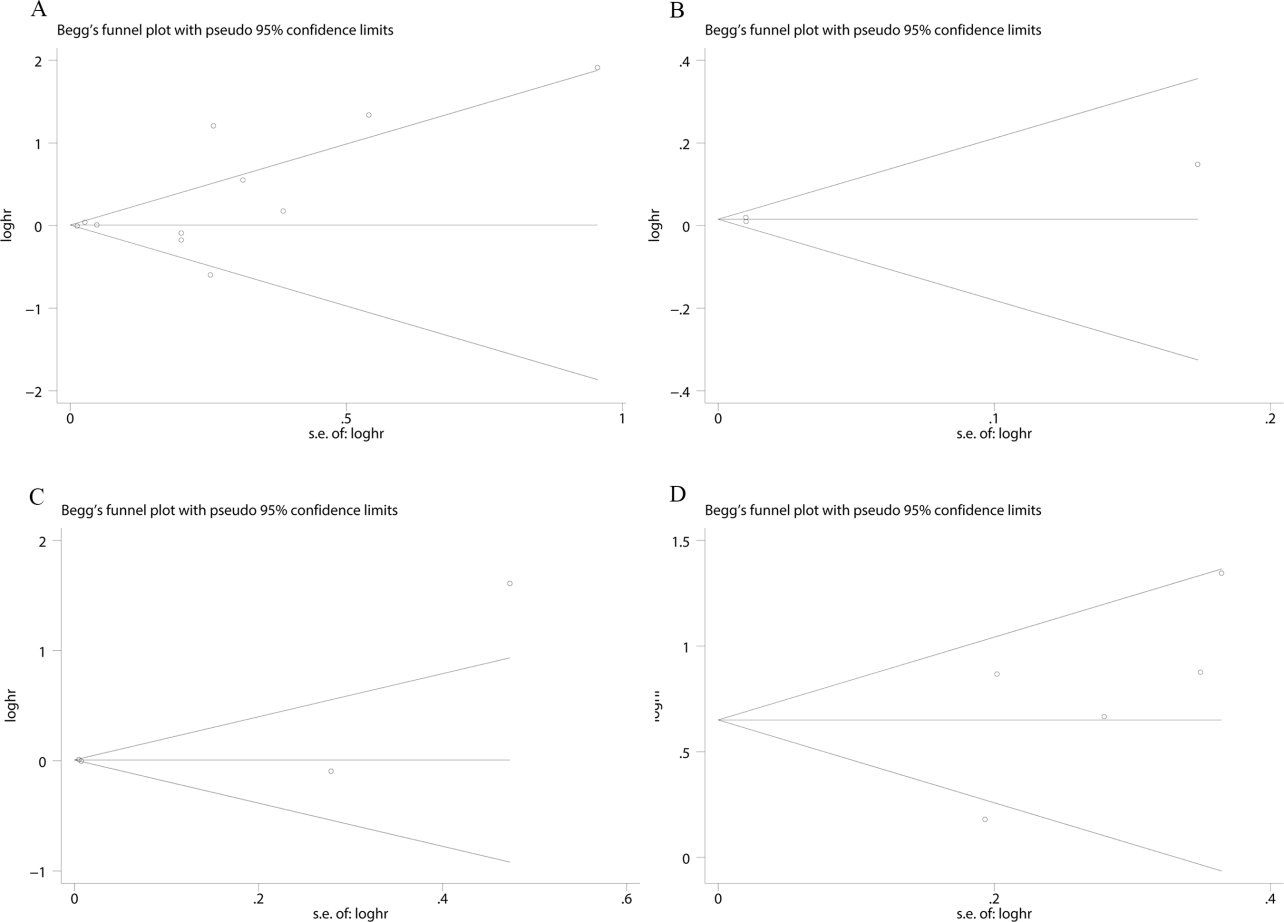
Begg's funnel plot evaluating the potential publication bias among the included studies (**A**). CD68 expression with RFS (*P* = 0.119); (**B**). CD68 expression with DSS (*P* = 1.0); (**C**). CD68 expression with OS (*P* = 0.734); (**D**). CD163 expression with RFS (*P* = 0.221).

To evaluate the publication bias of the included studies, Begg's and Egger's tests were performed. Begg's funnel plots showed no significant asymmetry among the included studies concerning the prognostic role of CD68^+^ TAMs with OS (*p* = 0.734) (Figure 5A), DSS (*P* = 1.0) (Figure 5B), and RFS (*P* = 0.119) (Figure 5C). As for the studies involved with the prognostic value of CD163^+^ TAMs with RFS, no significant publication bias was found by Begg's funnel plots (*P* = 0.221) (Figure 5D). The results derived from Egger's test were also consistent with abovementioned results (Supplementary Figure 3). Moreover, the results from “ trim and fill ” analyses also confirm our conclusions (detailed in Supplementary Figure 4) which suggest that potential missing data will not change the conclusion.

## DISCUSSION

Bladder cancer management is a thorny clinical problem because of frequent recurrence. TUR is performed as the primary treatment for NMIBC. However, most patients need to receive TUR repeatedly due to relapse. In order to reduce the rate of recurrence, BCG intravesical instillation as a type of immunotherapy was strongly recommended for patients diagnosed with carcinoma *in situ* (CIS), intermediate or high-risk NMIBC following TUR therapy. Recently, many studies have focused on other immunotherapy reagents. For example, Atezolizumab (Tecentriq™, MPDL3280A) as PD-L1/PD-1 immune checkpoint inhibitor, was approved as a second-line therapy for advanced bladder cancer in 2016 [23, 24]. Accumulating evidences have suggested that immune cells play prominent function in tumor microenvironment of bladder cancer and have great potential for bladder cancer management [6, 23].

In this meta-analysis, 11 out of the 13 included studies analyzed the role of TAMs in bladder cancer with CD68 marker, in combination with or without CD163 marker. 4 studies identified the TAMs with CD163 biomarker, of which 1 study evaluated the TAMs in the tumor stroma and islet with CD163 alone. we systemically reviewed and analyzed the prognostic value of TAMs identified by CD68 or CD163 biomarker in bladder cancer patients. Some of these studies suggested that TAM infiltration was significantly associated with poor survival in bladder cancer patients [10, 14, 15]. However, the pooled results derived from our meta-analysis showed no significant correlation between the density of CD68^+^ TAMs and OS, RFS, DSS and PFS in bladder cancer patients. Furthermore, subgroup analyses also indicated no significant association between elevated CD68^+^ TAMs density and RFS in bladder cancer patients, regardless of the CD68^+^ identified in whole sample area (or not specified), tumor stroma or tumor islet. However, our results indicated that elevated CD163^+^ TAMs density could predict poor RFS in the bladder cancer patients after TUR therapy. Subgroup analysis was performed with regard to the instillation drugs used after TUR, and the pooled results indicated that the high CD163^+^ TAMs density was associated with poor RFS in the bladder cancer patients treated with TUR followed by BCG instillation. Moreover, subgroup analyses also suggested elevated CD163^+^ TAMs density predicted poor RFS regardless of the CD163^+^ TAMs identified in various sample locations.

Traditionally, macrophages play the defensive roles against foreign pathogens and contribute to phagocytosis and presentation of antigens expressed on the surface of the apoptotic or infected cells [25, 26]. In the past two decades, emerging studies have indicated that macrophages can be educated in different stages of TME, and TAMs can play multifaceted function in immune surveillance and tumor development that correlated with specific expression of markers (immune phenotypes). In majority of previous studies, CD68 was used as a common biomarker for all macrophages in tumor samples. However, macrophages can polarize into different phenotypes, which are tightly regulated by tissue and tumor microenvironment [27, 28]. The most simplified classification of macrophages polarization were type 1 or inflammatory macrophages (M1) and type 2 or alternatively activated macrophages (M2) [29–31]. The general belief is that M1 macrophages induce inflammation that may contribute to tumor initiation. In contrast, M2 macrophages mediate tissue repair and immunosuppression that contribute to tumor progression [6, 32]. In most cases, M1 macrophages were characterized by expression of CD80, CD86, human leukocyte antigen (HLA)-DR, inducible nitric oxide synthase (iNOS), while M2 macrophages generally express markers such as CD163, CD204, CD206 and Arginase 1 [31, 32]. Future studies using these macrophage polarization markers may further characterize subpopulation of macrophages that associate with bladder cancer prognosis.

Current study is the first systemic review and meta-analysis to evaluate the prognostic value of CD68^+^ and CD163^+^ TAMs in bladder cancer. However, there are some limitations in our study. Firstly, heterogeneity among included studies cannot be completely avoided. Moreover, all the included studies were limited in English and Chinese language. Additionally, HRs and 95% CIs derived from multivariate analysis were generally extracted for this meta-analysis, however, Pichler's study only provided univariate HRs and 95% CIs [22], and the HRs and 95% CIs were extracted from the Kaplan–Meier survival curve in Suriano's study [15], which may overstate the prognostic role of TAMs in bladder cancer patients. Finally, the included studies and patients numbers were relatively limited. Therefore, future large scale studies may be required to validate that conclusion from our study.

In summary, current systemic review and meta-analysis draw a conclusion about the prognostic role of TAMs in bladder cancer patients. Our results suggested the elevated density of CD163^+^ TAMs predicted poor RFS in bladder cancer patients. While TAMs only identified with CD68 marker in bladder cancer samples were not significantly correlated with the prognostic outcomes and clinicopathological parameters in bladder patients. Due to the limitations in our study, further studies with larger sample size and rational design are still needed to validate our conclusion.

## MATERIALS AND METHODS

### Literature search strategy for relevant studies

This systemic review and meta-analysis was performed according to the Preferred Reporting Items for Systematic Reviews and Meta-Analyses statement (PRISMA) [33]. Related publications were retrieved from the database of Web of Science, Pubmed, Embase, Wanfang and China National Knowledge Infrastructure (CNKI). All the publications were searched by August 8, 2017 with the following keywords: “macrophage” and “bladder cancer” or “bladder carcinoma”. The references from retrieved articles were also manually assessed to avoid the omission.

### Studies selection

After removing duplications by Endnote X7, the remaining studies were reviewed by two authors independently, and disagreements were resolved by discussion and consensus. The inclusion criteria were as listed below: ① studies focusing on the prognostic value of TAMs infiltration in bladder cancer; ② TAMs were identified by immumohistochemical (IHC) staining in the bladder cancer samples; ③ with sufficient data to extract hazard ratios (HRs) and 95% confidence intervals (CIs). The exclusion criteria for the studies were in the following: ① duplicated studies; ② animal studies, case reports, comments, reviews and meeting abstracts; ③ studies not relevant with prognostic analyses; ④ studies without sufficient data to extract HRs and 95% CIs.

### Evaluation of the publications quality and data extraction

We evaluated the methodological quality of the included studies with the selection and outcome categories in the Newcastle-Ottawa Scale (NOS) system [34]. The evaluation was performed by two authors independently, and the disagreements were addressed by the discussion and consensus. After reviewing the full text of the included studies, we extracted the data with a predefined form including the following items: publication year, surname of the first author, country, cases number, tumor stages, therapy, and markers for macrophages, antibody source, sample locations, follow-up data and outcomes described in the studies. For the studies only with Kaplan-Meier survival curves, we extracted HRs and 95% CIs from the survival curves according to the methodology described by Tierney JF, et al [35] and via the Engauge Digitizer 4.1 software.

### Statistical analysis

Raw HRs and 95% CIs were extracted from all the included studies, then analysed with specified command provided by the Stata version 12.0 (Stata Corporation, Texas, USA), and the pooled results were presented with forest plots [36]. Heterogeneity of the included studies was described with Chi-squared and *p* value. I-square (I^2^) index was tested to evaluate the degree of total variation among the studies. I^2^ > 50% or *p* < 0.10 was defined as significant heterogeneous, and we preferred to choose random-effects model for analyses in this situation. Otherwise, we performed the meta-analysis using the fixed-effects model. The pooled results were considered significant if the 95% CIs did not include 1. If HRs were provided with results derived from both univariate and multivariate analyses in the same study, multivariate models were preferred for increased accuracy. In order to evaluate the influence of potential missing data, we further performed “ trim and fill ” analysis [37]. Begg's and Egger's tests were also performed to evaluate the publication bias among the included studies [38, 39].

The funder had no role in study design, data collection and analysis, decision to publish, or preparation of the manuscript.

## SUPPLEMENTARY MATERIALS FIGURES AND TABLES



## Abbreviations

TAMs: Tumor associated macrophages
IHC: immunohistochemistry
TME: tumor microenviroment
NMIBC: non-muscle invasive bladder cancer
MIBC: muscle invasive bladder cancer
BCG: Bacillus Calmette Guerin
TUR: transurethral resection of bladder tumor
RC: radical cystectomy
CD68^+^: CD68 positive
CD163^+^: CD163 positive
OS: overall survival
RFS: relapse-free survival
PFS: progression-free survival
CSS: cancer-specific survival
DSS: disease-specific survival
NOS: Newcastle-Ottawa Scale
HR: hazard ratio
OR: odds ratio
95% CI: 95% confidence interval
