# Supplementary material for: Prognostic roles of tumor associated macrophages in bladder cancer: a system review and meta-analysis
Source: Oncotarget. 2018 May 18;9(38):25294–303. doi: 10.18632/oncotarget.25334 (PMC5982745; doi:10.18632/oncotarget.25334)
Supplement: Supplementary file 1 [file oncotarget-09-25294-s001.pdf]

Prognostic roles of tumor associated macrophages in bladder cancer: a system review and meta-analysis

SUPPLEMENTARY MATERIALS

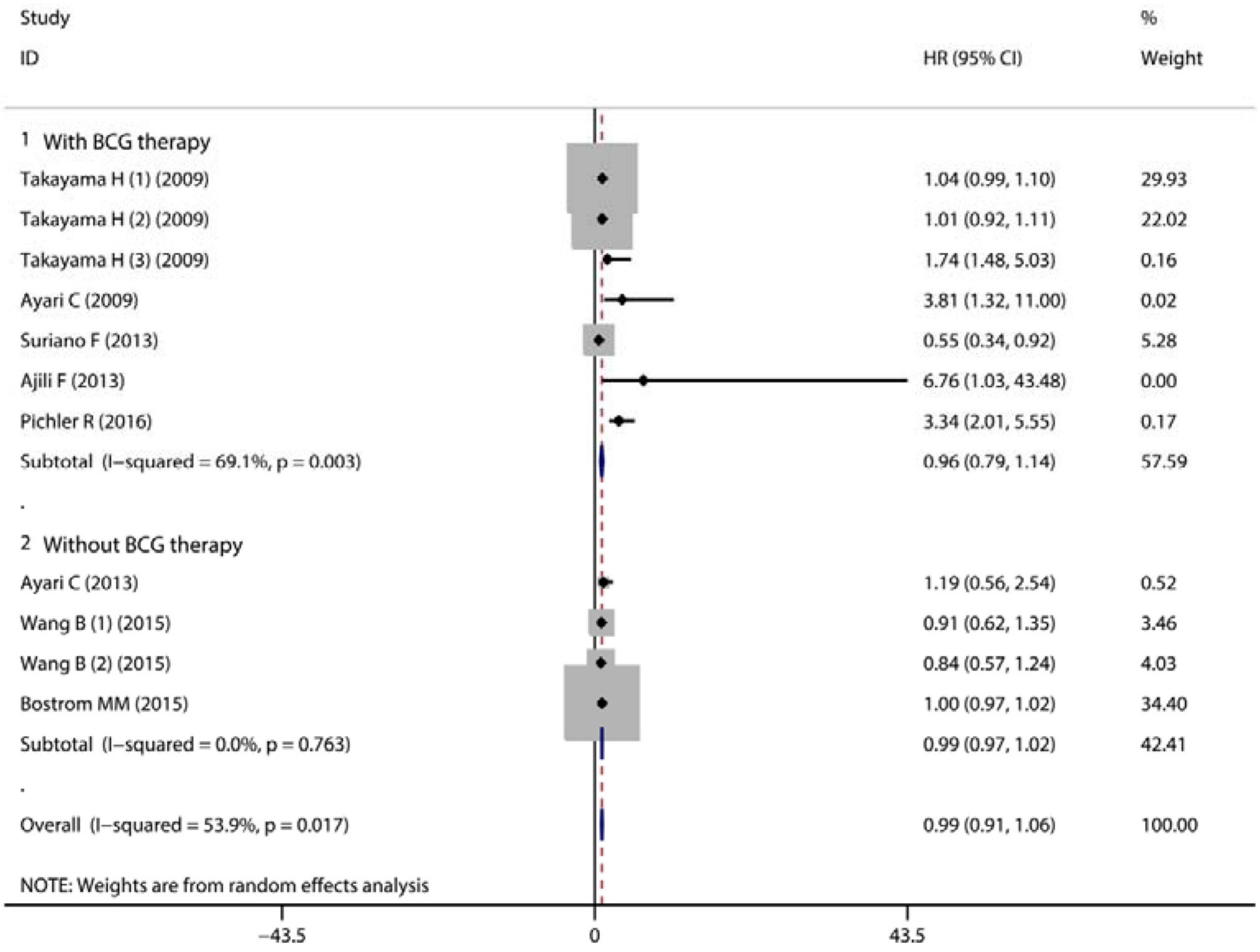

Supplementary Figure 1: Subgroup analyses concerning the prognostic role of CD68^{+} TAMs on RFS in bladder cancer patients with or without BCG therapy.

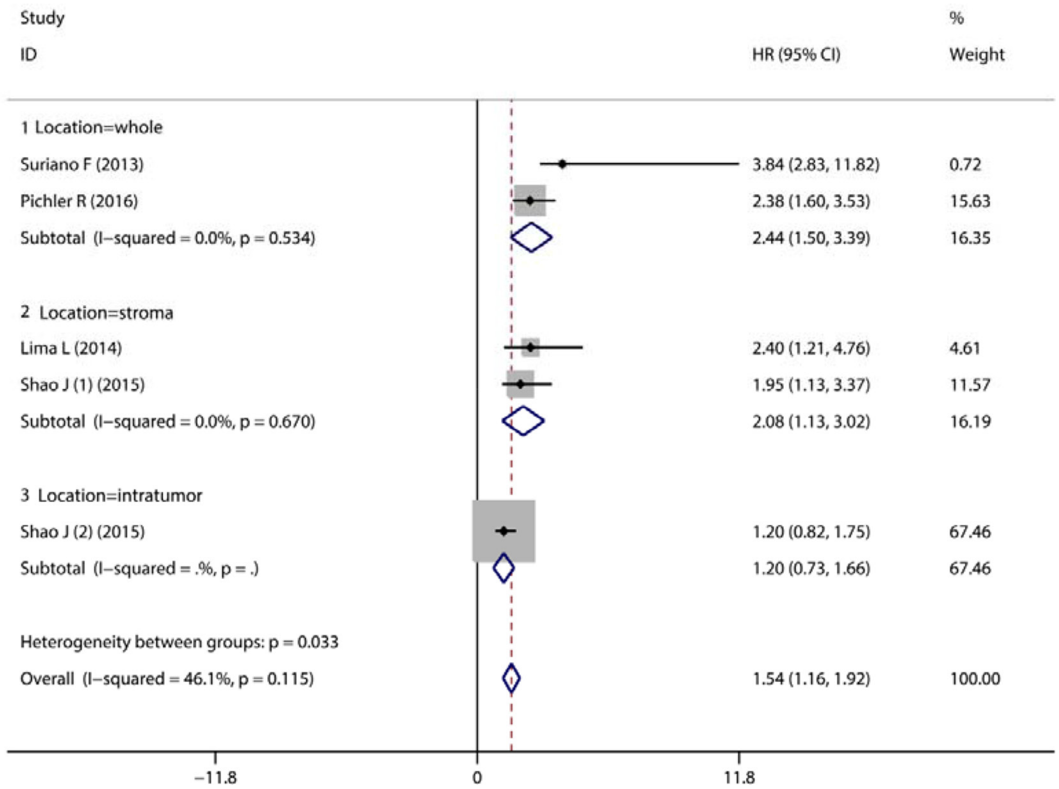

Supplementary Figure 2: Subgroup analyses concerning the prognostic role of CD163^{+} TAMs on RFS in bladder cancer patients with regard to different sample locations.

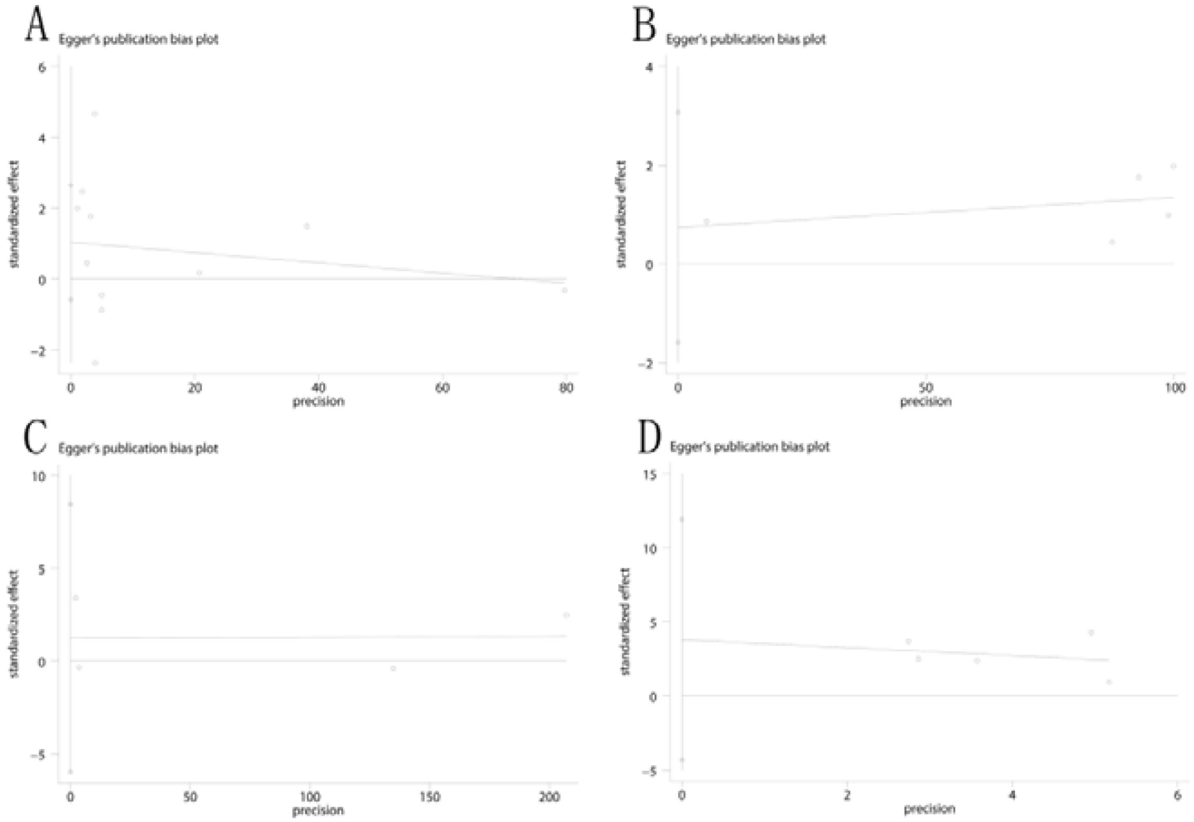

Supplementary Figure 3: Egger's test evaluating the potential publication bias among the included studies. (**A**). CD68 expression with RFS (P = 0.180); (**B**).CD68 expression with DSS (P = 0.472); (**C**). CD68 expression with OS (P = 0.538); (**D**). CD163 expression with RFS (P = 0.234).

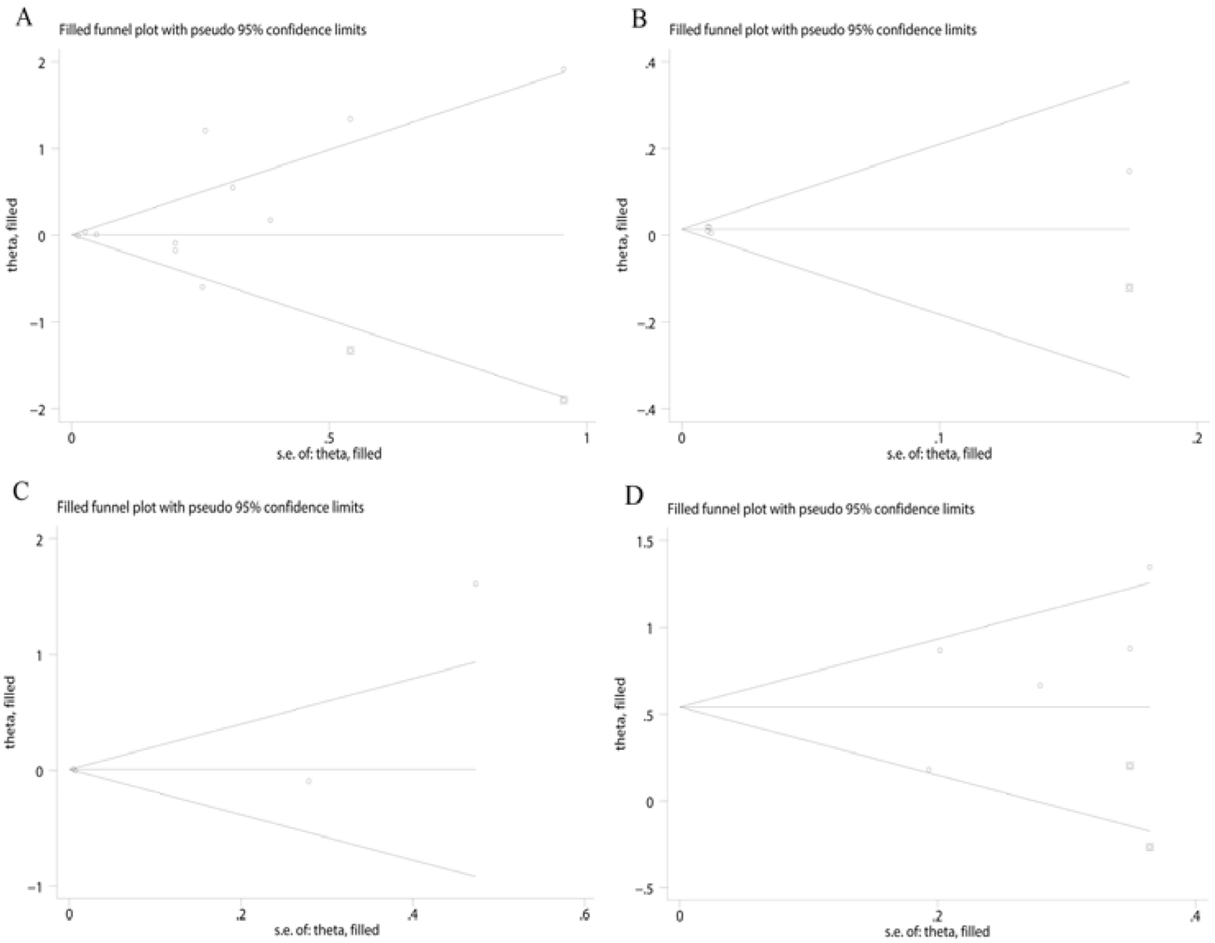

Supplementary Figure 4: "Trim and filled" analysis including the potential missing data. (**A**) CD68 expression with RFS; (**B**)CD68 expression with DSS; (**C**) CD68 expression with OS; (**D**) CD163 expression with RFS.
